# Association between Mir-17-92 gene promoter polymorphisms and depression in a Chinese population

**DOI:** 10.1186/s12920-024-01894-8

**Published:** 2024-05-06

**Authors:** Peng Liang, Xue Yang, Rui Long, Yue Li, Ziling Wang, Pingliang Yang, Yundan Liang

**Affiliations:** 1 Department of Pathology and Pathophysiology, School of Basic Medical Sciences, Chengdu Medical College, Chengdu, 610500 Sichuan P.R. China; 2 Department of Geriatric psychiatry, the First Special Hospital in Harbin, Harbin, P.R. China; 3 Department of Anesthesiology, The First Affiliated Hospital of Chengdu Medical College, Chengdu, P.R. China

**Keywords:** Depression, SNP, Mir-17-92 cluster, Genetic susceptibility

## Abstract

**Background:**

Depression is a common chronic debilitating disease with a heavy social burden. single nucleotide polymorphisms (SNPs) can affect the function of microRNAs (miRNAs), which is in turn associated with neurological diseases. However, the association between SNPs located in the promoter region of miR-17-92 and the risk of depression remains unclear. Therefore, we investigated the association between rs982873, rs9588884 and rs1813389 polymorphisms in the promoter region of miR-17-92 and the incidence of depression in a Chinese population.

**Methods:**

we used GWAS (Genome-wide association study) and NCBI (National Center for Biotechnology Information) to screen three SNPs in the miR-17-92 cluster binding sites. A case-control study (including 555 cases and 541 controls) was conducted to investigate the relationship between the SNPs and risk of depression in different regions of China. The gene sequencing ii was used to genotype the collected blood samples.

**Results:**

the following genotypes were significantly associated with a reduced risk of depression: rs982873 TC (TC vs. TT: OR = 0.72, 95% CI, 0.54–0.96, *P* = 0.024; TC/CC vs. TT: OR = 0.74, 95% Cl, 0.56–0.96, *P* = 0.025); CG genotype of rs9588884 (CG vs. CC: OR = 0.74, 95% CI, 0.55–0.98, *P* = 0.033; CG/GG vs. CC: OR = 0.75, 95% Cl, 0.57–0.98, *P* = 0.036); and AG genotype of rs1813389 (AG vs. AA: OR = 0.75, 95% CI, 0.57-1.00, *P* = 0.049; AG/GG vs. AA: OR = 0.76, 95% Cl, 0.59-1.00, *P* = 0.047). Stratified analysis showed that there was no significant correlation between the three SNPS and variables such as family history of suicidal tendency (*P* > 0.05).

**Conclusions:**

our findings suggest that rs982873, rs9588884, and rs1813389 polymorphisms may be associated with protective factors for depression.

## Introduction

Depression is a common chronic and debilitating disorder that is characterized by affective, cognitive, and behavioral symptoms [1]. Anhedonia, a reduced ability to experience pleasure or a lack of responsiveness to hedonic stimuli, is the core symptom of depression. According to the Institute for Health Metrics and Evaluation, anhedonia affects approximately 280 million people worldwide [2]. In its most severe form, depression can lead to suicide, which is a major cause of the increasing suicide rate in the 21st century [3, 4]. Depression is the result of a combination of social, psychological and biological factors. Although there are proven therapies for depression, more than 75% of people in low- and middle-income countries receive no treatment [5]. One factor hindering effective care is that people with depression are often not correctly diagnosed. Although there are a variety of hypotheses for the pathophysiological mechanism of depression, including inflammation [6], there is no clear mechanism that can comprehensively explain the pathogenesis. As a result, there are insufficient data to guide the diagnosis of clinical depression. Therefore, there is a need to identify markers to improve the monitoring, prognosis and therapeutic intervention for this serious global disease.

MicroRNAs (miRNAs) are a group of non-coding RNAs with a length of about 20 nucleotides encoded by the genome in cells. They negatively regulate gene expression at the post-transcriptional level by binding to the 3’-untranslated regions (3’-UTRs) of mRNA of target genes, leading to degradation or translational repression of mRNA [7]. miRNAs, which are relatively conserved in species evolution, participate in a variety of important biological processes including cell proliferation, differentiation and apoptosis [8]. Among various non-coding RNAs, miRNAs are the most studied and well characterized, and have emerged as major regulators of neuroplasticity and higher brain functions [9]. Previous studies have shown that overexpression or disorder of miRNAs is associated with the occurrence and development of many complex diseases [10], including neurological diseases such as Parkinson’s disease, Alzheimer’s disease and major depressive disorder [11]. Variations in specific miRNAs have potential as biomarkers for diagnostic or therapeutic targets in clinical practice [12]. However, the mechanisms by which miRNAs contribute to the development and progression of these neurological disorders, especially depression, remain largely unknown. Therefore, finding the specific miRNA and its potential mechanism may provide new diagnostic and therapeutic ideas for the treatment of depression.

In addition, it is worth noting that many miRNAs single nucleotide polymorphisms (SNPs) are associated with neurological diseases. For example, miR-5-2p rs41305272*T carrier frequency has been correlated with the number of anxiety and depressive disorders diagnosed per subject [13]. Another study demonstrated a significant association between miRNA-137 rs1625579 polymorphism and schizophrenia in a southern Chinese Han population [14]. Further results suggest that downregulation of miR-34b and miR-34c in the brain, and SNPs in the 3’-UTR of α-SYN, can increase α-SYN expression and may contribute to the pathogenesis of Parkinson’s disease [15]. Therefore, exploring the association between SNPs of specific miRNAs and depression is a new idea for studying the pathogenesis of depression.

Human miRNAs are characterized by clustering on chromosomes. These miRNA gene clusters are transcribed by a common promoter to generate polycistronic elements [16]. Among them, the miR-17-92 gene cluster located on chromosome 13 (13q31.3:91347820–91,354,575) encodes six mature miRNAs: namely miR-17, miR-18a, miR-19a, miR-20a, miR-19b-1 and miR-92a-1^16^. It has been found that miR-17-92 is highly expressed in the embryonic mouse brain [17] and alters the level of miR-17-92 in hippocampal neural precursor cells, which has important effects on neurogenesis and anxiety- and depression-related behaviors. Loss of miR-17-92 in neural progenitor cells results in decreased neurogenesis in the hippocampal dentate gyrus, while high expression increases neurogenesis [18]. miR-17-92 gene knockout mice show anxiety and depression-like behaviors, while miR-17-92 overexpression mice show anxiolytic and antidepressant behaviors [19]. These results suggest that the miR-17-92 cluster is a key regulator of hippocampal neurogenesis and anxiety and depressive behaviors [20], and therefore may be a new marker for the diagnosis of depressive disorders. However, the association between risk of depression and miR-17-92 SNPs, especially in the promoter region, has not been investigated.

Therefore, a case-control study was conducted to investigate the association between rs982873, rs9588884 and rs1813389 polymorphisms in the miR-17-92 promoter region and the incidence of major depressive disorder in a Chinese population; with the aim of providing a scientific basis for the prevention and treatment of depression.

## Materials and methods

### Subjects

We conducted a case-control study involving 555 patients with depression and 541 controls. Patients with depression were recruited from Jining Mental Hospital, Yunnan Mental Health Center, Sichuan Provincial People’s Hospital and Harbin First Specialized Hospital from September 2013 to April 2022. All patients were newly diagnosed with depression using the Fourth edition of the Diagnostic and Statistical Manual of Mental Disorders (DSM-IV) [21] and the Tenth Revision of the International Statistical Classification of Diseases and Related Health Problems (ICD-10) [22]. The recruited patients had a 24-item Hamilton Depression Scale (HAMD-24) total score of ≥ 24 before screening. Exclusion criteria included diagnosis of other psychiatric disorders, presence of neurological disorders, lack of signed consent, poor ability to participate in assessment, and pregnancy.

Healthy controls were randomly selected from individuals who participated in health checkups during the same period. Clinical data regarding the patient’s age, sex, pulse rate, age of onset, suicide attempt, whether the patient was the first episode, and family history were all derived from the medical record, which has been described in detail in our previous studies [23, 24]. The exclusion criteria for healthy controls were the same as for patients, and the mean age (206 men, 335 women) was 50 ± 18.1 years (Table 1). Controls and cases were frequency matched according to gender, age, and region of residence. Quanto software 1.2.3 (University of Southern California, Los Angeles, CA, USA) was used to calculate the power of sample size. When the RG value was set to 1.6, the heritability of the three SNPS was greater than 80% under the dominant model.

**Table 1:** Characteristics of the study population

Variables	Controls, *n* = 541	Case, *n* = 555
Age (years)	50 ± 18.1	42 ± 17.7
Gender (%)		
Male	206 (38.1)	160 (28.8)
Female	335 (61.9)	395 (71.2)
Age of onset (years)		37.1 ± 18.6
Pulse rate		79.4 ± 12.3
Depressive episode (%)		
Severe		278 (49.9)
Mild/moderate		277 (50.1)
Family history (%)		
Positive		97 (17.5)
Negative		458 (82.5)
Suicide attempt		
Yes		314 (56.6)
No		241 (43.4)
First episode (%)		
Yes		278 (50.1)
No		277 (49.9)

This study was approved by the Ethics Committee of Chengdu Medical College (approval number: Chengyi Ethics [2008] No. 15), and all procedures followed the principles of the Declaration of Helsinki. Written informed consent was obtained from each participant.

### SNP selection

The UCSC Genome Database [25] was searched to identify SNPs associated with depression in the 2 kb upstream of the transcription start site of the miR-17-92 promoter region. The loci with a minor allele frequency > 10% in the Asian population were selected as follows: rs982873, rs9588884 and rs1813389.

### Genotyping

Human blood collection: we collected 2-3 ml whole blood samples from all subjects using EDTA anticoagulated tubes and stored these on ice at -20℃.

DNA extraction: DNA was extracted from each peripheral blood sample using a whole blood genomic DNA rapid extraction kit (Sangon Bioengineering (Shanghai) Co., Ltd.) according to the instructions.

Multiple amplification and high-throughput sequencing: a primer pool containing three SNP sites were designed and synthesized. The primer sequences are shown in Table 2. The target SNP sequences were amplified, and Illumina sequencing libraries were prepared by two-step PCR. The first round PCR system was as follows: 2 µl DNA template (10 ng/µl); 1 µl upstream primer pool (10µM). The downstream primer pool (10µM) was 1 µl 2×PCR Ready Mix 15 µl (total volume 25 µl; Kapa HiFi Ready Mix). After preparation of the system, the following reaction program was performed on a PCR instrument (BIO-RAD, T100™): pre-denaturation at 98° C for 3 min, followed by eight cycles of denaturation at 98° C for 30 s, annealing at 50° C for 30 s, and extension at 72° C for 30 s. This was followed by 25 cycles of denaturation at 98° C for 30 s, annealing at 66° C for 30 s, and extension at 72° C for 30 s. The final extension was at 72° C for 5 min. After completion of the reaction, 4° C. At the end of the PCR, the correct size of the PCR products was confirmed by electrophoresis using 1% agarose gel, and the PCR products were recovered by purification using AMPure XP magnetic beads.

**Table 2:** Primers sequences used in this study

Description	Forward primer sequence (5’-3’)	Reverse primer sequence (5’-3’)
rs9588884	CAACCCCTTTCACTTATTTTTCAGC	GAAGGAGTACACTTCTTAGACTTGC
rs982873	TTCCAGAAACATCAACATTCCCAAA	ACAAGACTAGCCCTCTAGCAAAACA
rs1813389	AGGATTTCTCTAATGGGTCTAGAGT	TAAAGATTTTCGTACCCAGGGTAAG

A second round of PCR reactions was then performed using the first round of PCR products as templates to obtain libraries with molecular tags for sequencing. The reaction system was as follows: 2 µl DNA template (10ng/µl), 1 µl universal P7 primer (containing molecular tag, 10µM); Universal P5 primer (10µM) 1 µl; 2×PCR Ready Mix 15 µl (total volume 30 µl). After the reaction system was formulated, the following PCR procedure was performed: pre-denaturation at 98° C for 5 min, followed by five cycles of denaturation at 94 °C for 30 s, annealing at 55° C for 20 s, extension at 72° C for 30 s, and final extension at 72° C for 5 min. X. The final PCR products were recovered by purification using AMPure XP magnetic beads. Individual PCR products were mixed in equal amounts and sequenced using a HiSeq XTen sequencer (Illumina, San Diego, CA).

Data quality control and genotyping analysis: data quality control was performed by the following two steps: 1) Cutadapt (v1.2.1) software was used to cut out any part of the sequence containing the sequencing adapter; 2) PRINSEQ-lite (v0.20.3) software was used to control the quality of the remaining sequences, and the bases below the quality threshold of 20 were deleted according to the sequence from 3’ to 5’ end. The remaining sequences were regarded as qualified sequences. Then BWA (v0.7.13-r1126) software was used to align the qualified sequences to the reference genome with the default parameters. Based on the results, the genotypes of the target loci were calculated by SAMtools software (v0.1.18). Finally, ANNOVAR (2018-04-16) software was used for gene annotation. In addition, 5% of the samples were randomly selected for repeat genotyping for quality control, with 100% agreement for each SNP.

### Statistical analysis

All statistical analyses were performed using SPSS 25.0 (Chicago, IL, USA) and GraphPad Prism 5.0 (GraphPad Software, San Diego, CA, USA). The genotype frequencies of rs982873, rs9588884 and rs1813389 were calculated by direct counting. Hardy-Weinberg equilibrium was assessed by X [2] test. The distribution of rs982873, rs9588884 and rs1813389 genotypes in cases and controls was analyzed by X [2] test; and the association between the three polymorphisms and the risk of depression was evaluated by odds ratio (OR) and 95% confidence interval (Cl). A P value of < 0.05 was considered statistically significant.

## Results

The genotype frequencies of the three polymorphisms in controls and participants are shown in Table 3. The distribution of genotypes in the control group did not deviate from Hardy-Weinberg equilibrium (rs982873: *P* = 0.29; rs9588884: *P* = 0.33; rs1813389: *P* = 0.72). eQTL [26] query results showed no effect of rs982873, rs9588884 or rs1813389 polymorphism loci on the expression of miR-17-92. There was a significant difference between cases and controls in the following: the distribution of TC genotype of rs982873 (TC vs. TT: OR = 0.72, 95% CI, 0.54–0.96, *P* = 0.024; TC/CC vs. TT: OR = 0.74, 95% Cl, 0.56–0.96, *P* = 0.025); the distribution of rs9588884 CG genotype (CG vs. CC: OR = 0.74, 95% CI, 0.55–0.98, *P* = 0.033; CG/GG vs. CC: OR = 0.75, 95% Cl, 0.57–0.98, *P* = 0.036); the distribution of rs1813389 AG genotype (AG vs. AA: OR = 0.75, 95% CI, 0.57-1.00, *P* = 0.049; AG/GG vs. AA: OR = 0.76, 95% Cl, 0.59-1.00, *P* = 0.047).

**Table 3:** Association of the rs2242385, rs155979, rs3762983 and rs3762984 polymorphisms with depression risk

Models	Polymorphisms	Controls	Patients	Adjusted OR	*P*
		*N* = 541(%)	*N* = 555 (%)	(95%CI)	value
	rs982873				
Codominant	T/T	151 (27.9)	180 (32.4)	1.00	
	T/C	281 (52.0)	271 (48.8)	0.72 (0.54–0.96)	0.024
	C/C	109 (20.1)	104 (18.7)	0.80 (0.55–1.15)	0.230
Dominant	T/T	151 (27.9)	180 (32.4)	1.00	0.025
	T/C-C/C	390 (72.1)	375 (67.6)	0.74 (0.56–0.96)	
Recessive	T/T-T/C	432 (79.8)	451 (81.3)	1.00	0.940
	T/C	109 (20.1)	104 (18.7)	0.99 (0.73–1.35)	
	rs9588884				
Codominant	C/C	152 (28.1)	179 (32.2)	1.00	
	C/G	280 (51.8)	271 (48.8)	0.74 (0.55–0.98)	0.033
	G/G	109 (20.1)	105 (18.9)	0.82 (0.56–1.18)	0.280
Dominant	C/C	152 (28.1)	179 (32.2)	1.00	0.036
	C/G-G/G	389 (71.9)	376 (67.8)	0.75 (0.57–0.98)	
Recessive	C/C-G/G	432 (79.8)	450 (81.1)	1.00	0.990
	G/G	109 (20.1)	105 (18.9)	1.00 (0.73–1.36)	
	rs1813389				
Codominant	A/A	158 (29.2)	186 (33.5)	1.00	
	A/G	273 (50.5)	266 (47.9)	0.75 (0.57-1.00)	0.049
	G/G	110 (20.3)	103 (18.6)	0.82 (0.57–1.17)	0.270
Dominant	A/A	158 (29.2)	186 (33.5)	1.00	0.047
	A/G-G/G	383 (70.8)	368 (66.5)	0.76 (0.59-1.00)	
Recessive	A/A-A/G	431 (79.7)	452 (81.4)	1.00	0.840
	G/G	110 (20.3)	103 (18.6)	0.97 (0.71–1.34)	

CI Confidence interval, OR Odds ratio, OR was adjusted by age and gender

No significant association was found between rs982873, rs9588884 and rs1813389 polymorphisms and the following variables (*P* > 0.05; Table 4): depressive episode (severe vs. mild/moderate), family history (yes vs. no), first episode (yes vs. no).

**Table 4:** Stratified analyses of the rs982873, rs9588884, rs1813389 polymorphisms with depression risk

Variables	Frequency		OR (95% CI)	*P* value
	%	%		
rs1813389				
Depressive episode	Severe	Mild		
A/A	97 (34.9)	89 (32.1)	1.00 (Ref)	
A/G-G/G	181 (65.1)	188 (67.9)	0.88 (0.62–1.26)	0.500
Suicide attempt	Yes	No		
A/A	79 (32.8)	107 (34.1)	1.00 (Ref)	
A/G-G/G	162 (67.2)	207 (65.9)	0.95 (0.65–1.37)	0.770
First-episode patient	Yes	No		
A/A	91 (32.9)	95 (34.3)	1.00 (Ref)	
A/G-G/G	186 (67.2)	182 (65.7)	1.09 (0.76–1.56)	0.650
Family history	Yes	No		
A/A	25 (25.8)	161 (35.1)	1.00 (Ref)	0.056
A/G-G/G	72 (74.2)	297 (64.8)	1.60 (0.98–2.64)	
rs9588884				
Depressive episode	Severe	Mild		
C/C	93 (33.5)	86 (31.1)	1.00 (Ref)	
C/G-G/G	185 (66.5)	191 (69.0)	0.88 (0.62–1.27)	0.500
Suicide attempt	Yes	No		
C/C	103 (32.8)	76 (31.5)	1.00 (Ref)	
C/G-G/G	211 (67.2)	165 (68.5)	0.92 (0.63–1.34)	0.660
First-episode patient	Yes	No		
C/C	87 (31.7)	92 (33.2)	1.00 (Ref)	
C/G-G/G	190 (68.6)	185 (66.8)	1.08 (0.75–1.56)	0.660
Family history	Yes	No		
C/C	24 (24.7)	155 (33.8)	1.00 (Ref)	0.070
C/G-G/G	73 (75.3)	303 (66.2)	0.63 (0.38–1.05)	
rs982873				
Depressive episode	Severe	Mild		
T/T	94 (33.8)	86 (31.1)	1.00 (Ref)	
T/C-C/C	184 (66.2)	191 (69)	0.88 (0.61–1.26)	0.470
Suicide attempt	Yes	No		
T/T	105 (33.4)	75 (31.1)	1.00 (Ref)	
T/C-C/C	209 (66.6)	166 (68.9)	1.13 (0.78–1.65)	0.520
First-episode patient	Yes	No		
T/T	87 (31.4)	93 (33.6)	1.00 (Ref)	
T/C-C/C	190 (68.6)	184 (66.4)	1.11 (0.77–1.60)	0.560
Family history	Yes	No		
T/T	24 (24.7)	156 (34.1)	1.00 (Ref)	0.060
T/C-C/C	73 (75.3)	302 (65.9)	0.62 (0.38–1.03)	

CI Confidence interval, OR Odds ratio, OR was adjusted by age and gender

**Fig. 1:**
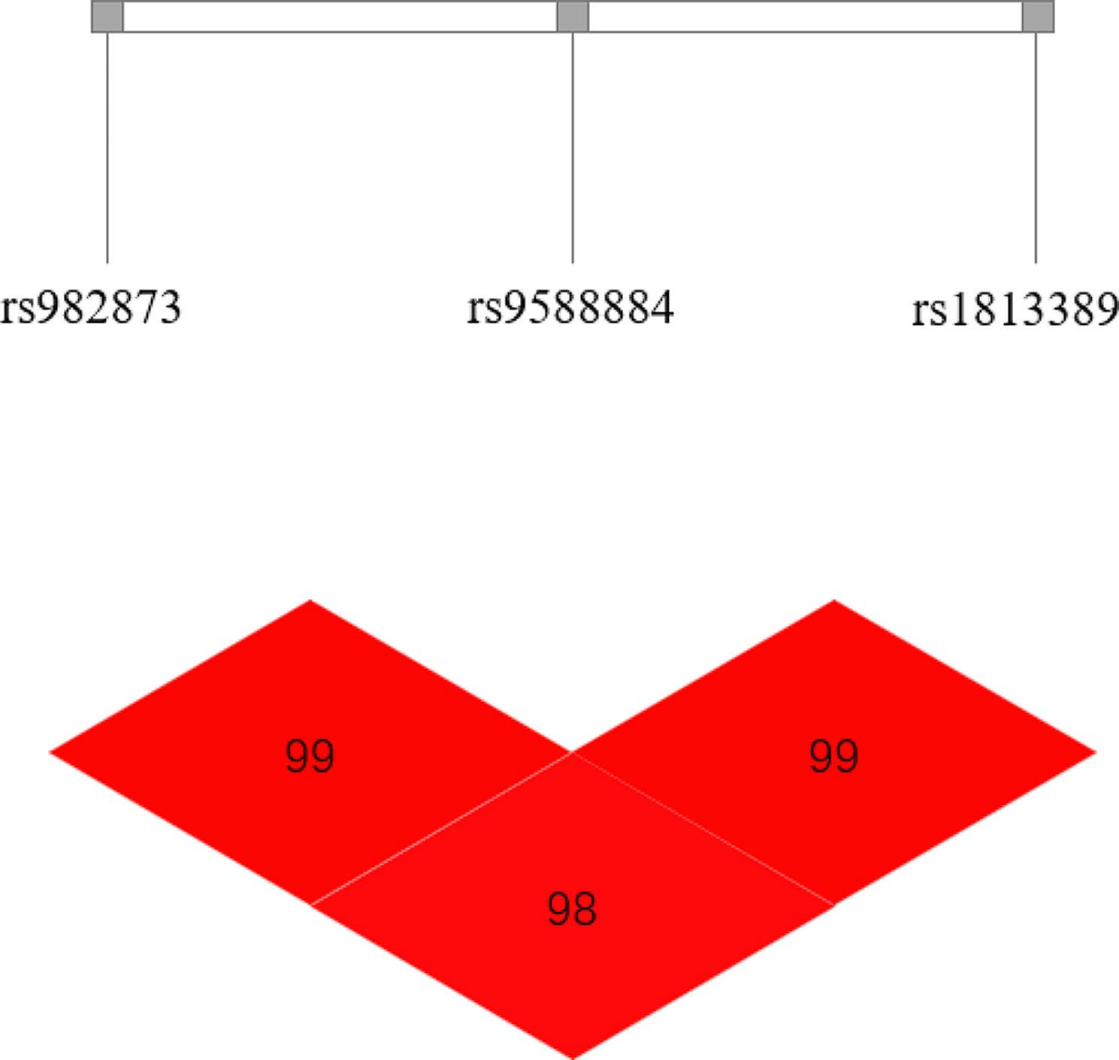
Linkage disequilibrium analysis of five SNP loci.

The haplotype analysis of rs982873, rs9588884 and rs1813389 polymorphism sites showed that rs982873, rs9588884 and rs1813389 had strong linkage disequilibrium (Fig. 1), and no significant difference was found between patients and healthy controls with different haplotype types (Table 5).

**Table 5:** Haplotype analysis of the rs982873、rs9588884、rs1813389 with the depression

Haplotype	Controls	Patients	OR (95% CI)	*P* value (Dimensionless)
	2 *N* = 1082(%)	2 *N* = 1100(%)		
AGC	8(0.7)	7(0.6)	1.000	
ACT	613(56.4)	593(53.6)	1.106(0.398–3.608)	0.847
GGC	459(42.2)	500(45.2)	1.245(0.448–3.460)	0.674

Haploid types with a population frequency lower than 0.05 were excluded from the study

## Discussion

Genetic variants, such as miRNAs or SNPs in miRNA promoter regions, can affect the regulation of miRNA-dependent gene expression. This is associated with a variety of diseases, including depression, and can alter an individual’s susceptibility to the disease; for examples microRNA processing genes DGCR8 rs3757 and AGO1 rs636832 are significantly associated with depression [27]. In our work, we used a case-control study design to investigate the relationship between miR-17-92 polymorphism and the risk of depression in a Chinese population for the first time. We found that the rs982873 TC, rs9588884 CG and rs1813389 AG genotypes were significantly associated with a reduced risk of depression. Our results suggest that the rs982873, rs9588884, and rs1813389 polymorphisms may be associated with protective factors for depression in this population. Since genetic polymorphisms rarely determine disease development, we can therefore only indicate changes in susceptibility or resistance to factors that contribute to the occurrence of the disease and/or the severity of its course [28]. This is because the phenotype depends not only on the genotype but also on its interaction with the environment. We also know that the interaction between genes and the environment is very complex. Because our bodies are exposed to many positive and negative factors [29], these can have an impact on the development of depression. However, we did not find an association between depressive symptoms and the studied polymorphisms, which may be due to individual differences.

There is growing evidence that depression is a complex and widespread disorder that affects thought, mood, and physical health. It is characterized by low mood, low energy, sadness, insomnia and an inability to enjoy life. However, it is often difficult to diagnose, especially in primary medical care [30]. At present, the incidence of depression in students is increasing year by year, and the incidence is high in young group [31]. Depression can also be misdiagnosed and, in addition, detection of mild depression is difficult because symptoms can be confused with symptoms of nervousness under stress [31]. Therefore, finding rapid tests to assess the risk of depression is necessary to complement a medical diagnosis. Biomarkers such as gene mutations, neurotransmitters and cytokines have been widely used in clinical practice to identify diseases. Our study showed that rs982873, rs9588884 and rs1813389 polymorphisms in the miR-17-92 promoter region were associated with the risk of depression and therefore may be potential biomarkers for a diagnosis.

The genetic variation of miRNA is closely related to various human diseases, and may lead to the risk of diseases in different systems. The miR-17-92 cluster is known to play an important role in multiple systems such as digestion, circulation and immunity. The miR-17-92 cluster has been found to affect nervous system development, and is associated with a variety of neurological diseases. One study suggested that the miR-17-92 cluster could enhance neuroplasticity and functional recovery after stroke in rats [32]. Another paper found that it can regulate neural and vascular regeneration in the adult CNS [33]. A further study demonstrated that it regulates multiple functionally relevant voltage-gated potassium channels in chronic neuropathic pain [34], and another showed that miR-19 plays an important role in glioma pathogenesis [20]. One other study found that downregulation of miR-17-5p expression contributes to PQ-induced dopaminergic neurodegeneration [35]. The results of the above studies all play a guiding role in our study. However, to the best of our knowledge, genetic variation in miR-92-17 has not been investigated in depression. In this study, we evaluated the association between SNPs located in the promoter region of the miR-17-92 cluster and depression in a Chinese population. Our results showed that rs982873 TC, rs9588884 CG and rs1813389 AG genotypes were significantly associated with reduced risk of depression.

Previous studies on genetic polymorphism and depression mostly focused on the coding region of genes, and there are relatively few studies on the non-coding region. Our study is a pioneering exploration since it includes a relatively large sample from different regions of China, which increases the accuracy of our results. However, our study also has some limitations. We cannot explain the effects of rs982873, rs9588884 and rs1813389 polymorphisms on gene expression and function. In this study, the whole genomic DNA of peripheral blood was extracted for the study, and the influence of other components of blood on the results was not considered. These questions will be refined in future experiments.

## Data Availability

The datasets generated and/or analyzed in the current study are not publicly available due to authors’ concerns about leaking data from the paper, but are available to the corresponding authors upon reasonable request.
